# Three-dimensional (3D) augmented reality during Retzius-sparing robot-assisted radical prostatectomy (RS-RARP): First experience

**DOI:** 10.1016/j.cpt.2024.06.003

**Published:** 2024-06-20

**Authors:** Silvia Secco, Alberto Olivero, Stefano Tappero, Paolo Dell’Oglio, Luca Carbonaro, Alessandro Marando, Angelo Vanzulli, Emanuela Bonoldi, Aldo Massimo Bocciardi, Antonio Galfano

**Affiliations:** a Department of Urology, ASST Grande Ospedale Metropolitano Niguarda, Milan 20162, Italy; b Department of Urology, Netherlands Cancer Institute-Antoni Van Leeuwenhoek Hospital, Amsterdam 1066, the Netherlands; c Interventional Molecular Imaging Laboratory, Department of Radiology, Leiden University Medical Center, Leiden 2333, the Netherlands; d Department of Radiology, ASST Grande Ospedale Metropolitano Niguarda, Milan 20162, Italy; e Department of Hematology, Oncology, and Molecular Medicine, ASST Grande Ospedale Metropolitano Niguarda, Milan 20162, Italy

**Keywords:** Augmented reality, Imaging, Three-dimensional, Prostatectomy, Robot surgery

Implementing augmented reality (AR) and three-dimensional (3D) imaging within routine surgical practice is challenging. This requires considerable time, investment, and perseverance. Recently, the application of such technologies has been scrutinized within the context of urology across multiple settings, including preoperative planning, intraoperative diagnostics, training, and counseling.^1^ Specific attention has been paid to standard anterior robot-assisted radical prostatectomy (RARP), where AR has demonstrated promising results. Studies have shown it to be feasible and reproducible,^2^ providing accurate identification of prostatic lesions and playing a pivotal role in minimizing positive surgical margins (PSMs).^3^

Conversely, there is a notable gap in the literature regarding the application of AR to Retzius-sparing RARP (RS-RARP). RS-RARP reportedly offers comparable oncological outcomes, specifically in terms of PSMs, and enhanced functional results, particularly with regard to urinary continence recovery up to 12 months postoperatively.^4^

In the current preliminary study, we took a step forward in addressing the aforementioned lack of evidence by analyzing the performance of AR during RS-RARP for the first time. Further studies will either confirm or refute our initial findings.

The current study included patients with prostate cancer (PCa) treated with RS-RARP at the ASST Grande Ospedale Metropolitano Niguarda (Milan, Italy) between April and October 2021. Each patient's diagnosis was based on a fusion biopsy targeted to one multiparametric magnetic resonance imaging (mpMRI) index lesion, classified according to the Prostate Imaging-Reporting and Data System (PI-RADS) as PI-RADS ≥4. Absence of mpMRI, poor quality of mpMRI, PI-RADS ≤3 mpMRI lesions, or lesions in the transitional zone represented the exclusion criteria. Nine patients were enrolled in this preliminary study.

Two dedicated uroradiologists reviewed mpMRI performed outside of our institutional radiology department.

The Digital Imaging and Communications in Medicine (DICOM) images were processed by Medics Srl (www.medics3d.com, Turin, Italy) to produce the Hyper Accuracy 3D (HA3D™) virtual models [Figure 1], which were used for preoperative planning and intraoperative support.

**Figure 1:**
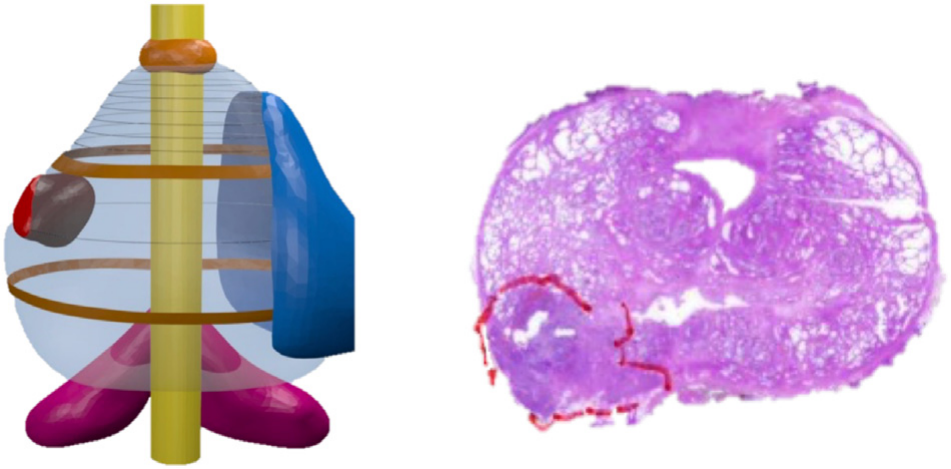
Hyper Accuracy three-dimensional (HA3D™; Medics Srl, Turin, Italy) virtual model and histopathological correspondence in the specimen. Each anatomical part of interest was reconstructed using dedicated medical-grade software (Mimics Medical 21.0; Materialise, Leuven, Belgium). Bioengineers have segmented the prostate gland, urethra, neurovascular bundles (NVBs), sphincter, seminal vesicles, and prostate cancer lesions using contour-based methods and computer vision tools. This process allowed for a good correspondence between the histopathological specimens. In the figures, the reader can observe the 3D model based on MRI; the PI-RADS >4 region is highlighted in red. The pathological section of the specimen is shown on the right, with correspondence between location and extension. 3D: Three-dimensional; HA3D™: Hyper Accuracy three-dimensional; MRI: Magnetic resonance imaging; NVB: Neurovascular bundle; PI-RADS: Prostate Imaging-Reporting and Data System.

All patients underwent RS-RARP as previously described,^5^ using a four-arm DaVinci Xi Surgical System (Intuitive Surgical Inc., Sunnyvale, CA, USA). Four expert robotic surgeons (AG, GP, SS, and PDO) performed the procedure. Each RS-RARP procedure was performed using the HA3D model superimposed over an endoscopic anatomical view [Figure 2]. A biomedical engineer manually performed the real-time overlapping of the HA3D model on the prostate during the procedure. The resulting mixed-stream video was returned to the DaVinci console using TilePro™ multi-input display technology (Intuitive Surgical Inc., Sunnyvale, CA, USA). The site of the lesion, once identified by the overlapping HA3D model, was marked with a stitch placed on the prostate.

**Figure 2:**
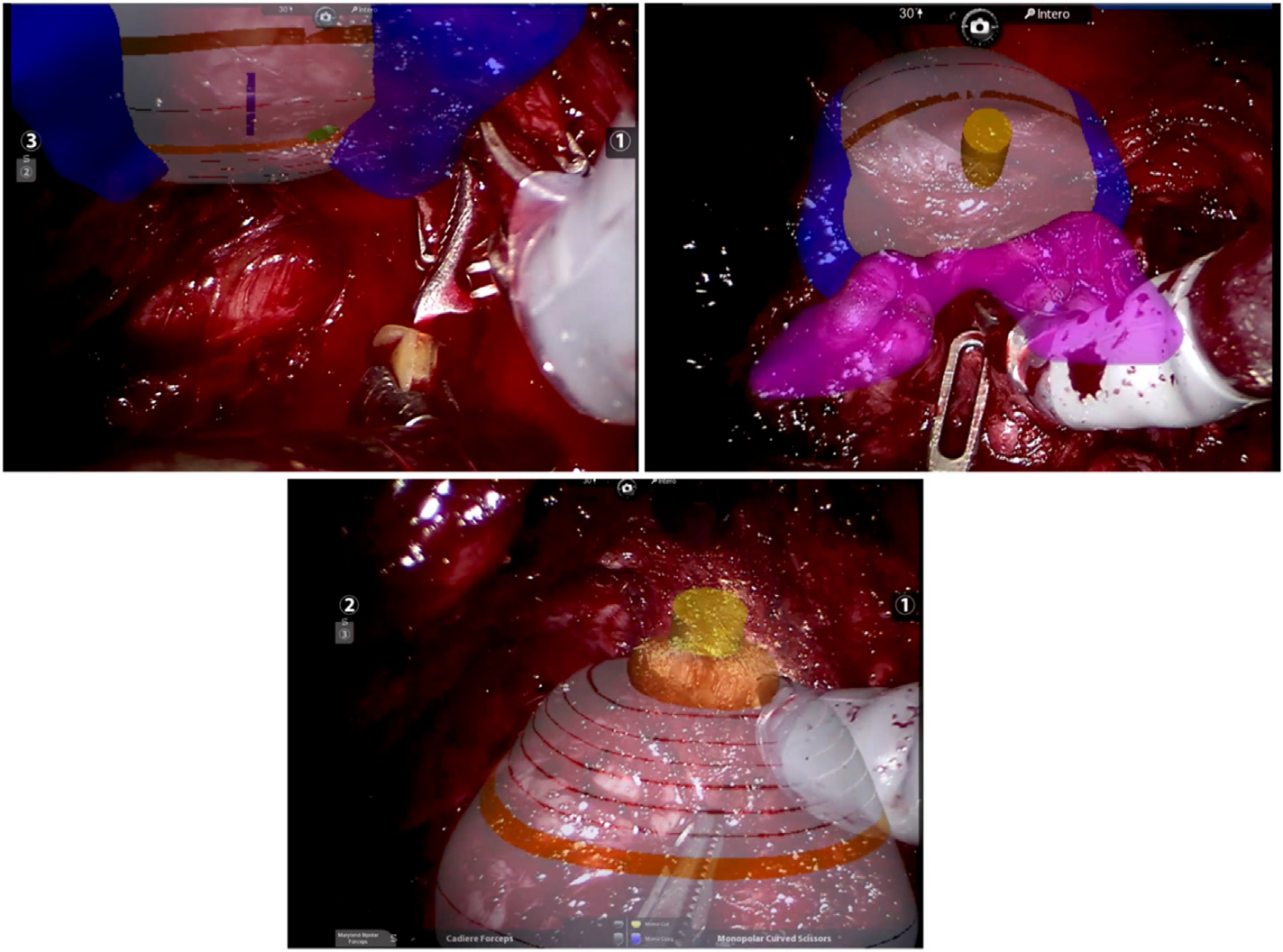
Overlay in highlights of surgery. Through the ICON™ software (Medics Srl, Turin, Italy), the Hyper Accuracy three-dimensional model could be overlayed over the endoscopic view. Specifically, the parts could be zoomed, rotated, and translated to align with the model based on the surgeon's needs. The transparency of the prostate gland could be adjusted to visualize the lesion. In the three reported figures, we can observe the adaptation of the model in different surgical phases (prostatic bundles isolation, prostate mobilization with Cadiere forceps, and urethra dissection).

The median (range) age, prostate volume, and prostate-specific antigen (PSA) of the enrolled patients were 61.6 (51–69) years, 46.1 (27–80) mL, and 4.9 (1.2–13) ng/mL, respectively. On mpMRI, six patients (67%) presented with PI-RADS 4 lesions, and three (33%) presented with PI-RADS 5 lesions. The median lesion diameter was 12 (5–25) mm, and no lesions were found in the transition zone. At the final pathology, two (22%), one (11%), and six (67%) patients harbored the pT2a, pT2b, and pT2c stages, respectively. In six patients (67%), one additional lesion was detected beside the index lesion targeted at the fusion biopsy and AR.

Correspondence of PCa location between HA3D models and final pathology was recorded in seven (78%) cases. In the remaining two (22%) cases, stitches were placed 2 and 3 mm from the nodule [Table 1].

**Table 1:** Summary of the results for the nine patients in the study (*n* = 9).

Variables	Value
Age (years)	61.6 (51–69)
Prostate volume (mL)	46.1 (27–80)
PSA level (ng/mL)	4.9 (1.2–13)
PI-RADS 4 lesions	6 (67)
PI-RADS 5 lesions	3 (33)
Lesion diameter (mm)	12 (5–25)
pT2a stage at final pathology	2 (22)
pT2b stage at final pathology	1 (11)
pT2c stage at final pathology	6 (67)
Correspondence of PCa location between HA3D™ models and final pathology	7 (78)

Values are expressed as *n* (%) or median (range). HA3D™: Hyper Accuracy three-dimensional; PCa: Prostate cancer; PI-RADS: Prostate Imaging-Reporting and Data System; PSA: Prostate-specific antigen.

AR in surgical procedures, particularly RARP, is emerging as a significant advancement that enables surgeons to visualize anatomical structures with enhanced precision. This technological leap provides a tangible overlay of crucial data from the surgeon's perspective, resulting in improved decision-making during complex procedures. The integration of this cutting-edge technology has demonstrated promising potential for transforming preoperative staging, simulating surgical interventions, and refining intraoperative strategies using 3D modeling. Studies conducted by Porpiglia et al.^2^^,^^3^^,^^6^ have provided an empirical foundation for the utility of AR, demonstrating the strength of the technology in accurately identifying tumor locations in real-time. Similarly, Schiavina et al.^7^ demonstrated that AR improved real-time identification of the index lesion, allowing the nerve-sparing approach to be changed in approximately one-third of cases, with 94.4% overall appropriateness. Furthermore, AR has been used to guide intraoperative frozen sections for nerve-sparing during RARP and has proven to be useful.^8^ Additionally, 3D-printed models are helpful in educating patients about their anatomy, disease, tumor characteristics, and surgical procedures.^9^

However, the use of AR during RS-RARP requires adequate analysis. We aim to present our preliminary results using HA3D models in RS-RARP at a high-volume institution, focusing on the correspondence of PCa location between AR and the final pathology.

Despite the enthusiasm around AR's capabilities, its application in RS-RARP is still in its nascent evaluation stages. RS-RARP is gaining popularity among urological surgeons globally owing to its impressive postoperative functional outcomes. The exploration of AR in this innovative surgical technique is timely and may be pivotal for optimizing surgical accuracy and enhancing patient prognosis.^10^

Our study performed a critical analysis of AR deployment in RS-RARP procedures at a prominent medical institution, focusing on the correlation between the AR-assisted preoperative identification of PCa and pathological outcomes. We discovered that AR is feasible but comes with challenges. The need for an engineer to direct AR's functions during surgery introduces complexity to the procedure, underscoring its significant dependence on technology.^11^ The surgeon must instruct the engineer on how to impose, rotate, and stretch the model during the different steps of the procedure, which is time-consuming and distracting. Therefore, a learning curve is required for both engineers and surgeons.

Our observations align with those of other authors who have previously pointed out issues with this technology. Creating the model requires time, and the engineer's skills in reconstruction and manual rotation of the model during surgery are highly dependent on correctly matching it. This can also distract the surgeon, thereby slowing the surgical flow. Thus, Pokorny and Yaxley^12^ stated that AR is “not yet ready for prime time”. The reconstruction process is also expensive, and whether the real clinical benefits justify the technology cost of USD >1000 per case is yet to be demonstrated.

Despite these limitations, we believe that AR has the potential to improve surgical proficiency and patient outcomes and revolutionize how RS-RARP is taught and learned. We anticipate that future robotic platforms with incorporated software will follow the traction of tissues and compensate for deformations caused by their elasticity, which will help identify tumor lesions and neurovascular bundles, reduce surgical margins, and improve functional outcomes. Further studies should aim to investigate the PSM rate when AR is used because a 3D model could help surgeons avoid PSM during RS-RARP.

Our study had some limitations due to its small sample size. Statistical analyses could not be performed, and robust conclusions could not be drawn. Moreover, the lack of comparisons with patients undergoing AR-guidance-naïve RS-RARP impedes our understanding of the impact of AR on surgical duration, adverse events, and lesion detection accuracy. Lastly, all surgeons involved in the study are expert MRI readers, which could be a confounding factor in evaluating the real benefits of AR during the procedure. However, these limitations are not unique to our study, as previous studies on innovative technical refinements share these limitations.

Albeit preliminary, our findings suggest that HA3D models may assist surgeons in locating the site of PCa during RS-RARP. Further efforts are required to address issues related to manual image superimposition, enhance the accuracy of the models, and make the availability of such technology more democratic.

## Authors contribution

Study concept and design: Silvia Secco, Antonio Galfano, Angelo Vanzulli, Emanuela Bonoldi, Aldo Massimo Bocciardi, and Alberto Olivero; Acquisition of data: Paolo Dell’Oglio, Alberto Olivero, Luca Carbonaro, and Alessandro Marando; Analysis and interpretation of data: Silvia Secco and Stefano Tappero; Drafting of the manuscript: Silvia Secco, Alberto Olivero, and Stefano Tappero; Critical revision of the manuscript for important intellectual content: Aldo Massimo Bocciardi, Antonio Galfano, Angelo Vanzulli, and Emanuela Bonoldi; Statistical analysis: Silvia Secco; Supervision: Antonio Galfano and Aldo Massimo Bocciardi. All the authors have read and approved the final version of the manuscript.

## Ethics statement

All procedures involving human participants were conducted in accordance with the 1964 *Declaration of Helsinki* and its later amendments or comparable ethical standards, and were approved by the Institutional Review Board (No. 3337/1900). Written informed consent was obtained from all participants.

## Declaration of generative AI and AI-assisted technologies in the writing process

In the writing process, we used AI-assisted technologies to improve readability and language. After using this tool/service, the author(s) reviewed and edited the content as needed and take(s) full responsibility for the content of the publication.

## Funding

None.

## Data availability statement

The datasets used in the current study are available from the corresponding author on reasonable request.

## Conflict of interest

The authors declare that they have no known competing financial interests or personal relationships that could have appeared to influence the work reported in this paper.

## References

[1] Rodler S., Kidess M.A., Westhofen T. (2023). A systematic review of new imaging technologies for robotic prostatectomy: from molecular imaging to augmented reality. J Clin Med.

[2] Porpiglia F., Fiori C., Checcucci E., Amparore D., Bertolo R. (2018). Augmented reality robot-assisted radical prostatectomy: preliminary experience. Urology.

[3] Porpiglia F., Checcucci E., Amparore D. (2019). Three-dimensional elastic augmented-reality robot-assisted radical prostatectomy using hyperaccuracy three-dimensional reconstruction technology: a step further in the identification of capsular involvement. Eur Urol.

[4] O'Connor-Cordova M.A., Macías A.G.O., Sancen-Herrera J.P. (2023). Surgical and functional outcomes of Retzius-sparing robotic-assisted radical prostatectomy versus conventional robotic-assisted radical prostatectomy in patients with biopsy-confirmed prostate cancer. Are outcomes worth it? Systematic review and meta-analysis. Prostate.

[5] Dell'Oglio P., Tappero S., Longoni M. (2022). Retzius-sparing robot-assisted radical prostatectomy in high-risk prostate cancer patients: results from a large single-institution series. Eur Urol Open Sci.

[6] Porpiglia F., Checcucci E., Amparore D. (2019). Augmented-reality robot-assisted radical prostatectomy using hyper-accuracy three-dimensional reconstruction (HA3D^TM^) technology: a radiological and pathological study. BJU Int.

[7] Schiavina R., Bianchi L., Lodi S. (2021). Real-time augmented reality three-dimensional guided robotic radical prostatectomy: preliminary experience and evaluation of the impact on surgical planning. Eur Urol Focus.

[8] Bianchi L., Chessa F., Angiolini A. (2021). The use of augmented reality to guide the intraoperative frozen section during robot-assisted radical prostatectomy. Eur Urol.

[9] Wake N., Rosenkrantz A.B., Huang R. (2019). Patient-specific 3D printed and augmented reality kidney and prostate cancer models: impact on patient education. 3D Print Med.

[10] Tappero S., Vecchio E., Palagonia E. (2023). Retzius-sparing robot-assisted radical prostatectomy after previous trans-urethral resection of the prostate: assessment of functional and oncological outcomes. Eur J Surg Oncol.

[11] Secco S., Galfano A., Barbieri M. (2019). Technical features and the demonstrated advantages of the Retzius sparing robotic prostatectomy. Arch Esp Urol.

[12] Pokorny M., Yaxley J. (2019). Three-dimensional elastic augmented reality for robot-assisted laparoscopic prostatectomy: pushing the boundaries, but cutting it fine. Eur Urol.

